# The development of the visual screening tool for anxiety disorders and depression: Addressing barriers to screening for depression and anxiety disorders in hypertension and/or diabetes

**DOI:** 10.4102/phcfm.v10i1.1721

**Published:** 2018-06-19

**Authors:** Zimbini Ogle, Liezl Koen, Dana J.H. Niehaus

**Affiliations:** 1 Department of Psychiatry, Stellenbosch University, South Africa; 2 Stikland Psychiatric Hospital, Bellville, South Africa

## Abstract

**Background:**

There is a lack of screening tools for common mental disorders that can be applied across cultures, languages and levels of education in people with diabetes and hypertension.

**Aim:**

To develop a visual screening tool for depression and anxiety disorders that is applicable across cultures and levels of education.

**Setting:**

Participants were purposively recruited from two not-for-profit organisations and two public health facilities – a maternal mental health unit and a primary health care centre.

**Method:**

This was a qualitative cross-sectional study. Thirteen drawings based on the Hospital Anxiety and Depression Scale depicting symptoms of anxiety disorders and depression were drawn. Participants described emotions and thoughts depicted in the drawings. Data were analysed through content analysis.

**Results:**

Thirty-one women (66%) and 16 men (34%) participated in the development of the visual screening tool. The mean age was 34 (standard deviation [SD] 12.46). There were 32 (68%) black participants, 11 (23%) mixed race participants and 4 (9%) white participants. Two participants (4%) had no schooling, 14 (31%) primary schooling, 8 (18%) senior schooling, 13 (29%) matric qualification and 8 (18%) had post-matric qualification. Participants correctly described 10 out of the 13 visual depiction of symptoms as associated with depression and anxiety disorders, with no differences between levels of education and cultural groups.

**Conclusion:**

Ten drawings were appropriate for inclusion in the visual screening tool for anxiety disorders and depression (VISTAD). The VISTAD will be validated against the mini international neuropsychiatric interview (MINI) in a primary care population with hypertension and/or diabetes.

## Introduction

Diabetes and hypertension are often comorbid with depression and/or anxiety disorders.^1,2,3,4,5^ This comorbidity with depression and anxiety disorders has been shown to decrease adherence to treatment regimens,^6^ increase rates of poor quality of life,^7^ increase mortality risk^8^ and inflate financial burden associated with health care.^9,10^ In spite of this co-existence and its effects being well known, mental disorders in patients with chronic physical illness largely remain undetected and untreated at primary health care.^11,12,13,14^ Lecrubier^15^ argues that the detection and treatment of depression and anxiety disorders can address the burden imposed by these disorders.

Jenkins et al.^16^ and Van Oers and Schlebusch^17^ identified the lack of appropriate screening tools as one of the barriers in detecting mental health problems. Multicultural societies, such as South Africa with its 11 official languages, lack screening tools that can be applied to a diverse range of cultural and language groups.^18^ A number of screening tools fail to meet acceptability for sensitivity and positive predictive value in the South African population^19^ and cannot be generalised to populations different from those who participated in their development.^20^

Screening tools are often translated into other languages for use by other cultural groups. In making screening tools available for use in people across different languages, research has documented the loss of meaning in translation.^21,22^ Steele and Edwards^23^ argue that the translation process is often plagued by practical and methodological difficulties that threaten the validity of the cross-cultural research projects. Hermanns et al.^24^ argue that the translation of concepts across cultures is crucial in order to develop culturally appropriate measurement tools, diagnoses and services for people with depression and anxiety disorders. Language, according to Alexander et al.,^25^ is a factor that contributes to the non-detection of mental health problems.

Educational status has been highlighted as a critical variable to be considered when developing a screening tool.^18^ Screening tools for depression and anxiety disorders have been found to be less effective in individuals with lower levels of education.^24,26,27^

Researchers have developed screening tools using drawings for example, Puertas et al.^28^ developed the FACES test which consisted of schematic faces representing mood states ranging from happy to sad face, and from one extreme to the other. The FACES test, according to Puertas et al.^28^ is a visual analog scale representation of mood, consisting of seven graded faces from happiest mood to saddest mood. Puertas et al observed that participants with no education were significantly more likely not to be able to answer the FACES test.

Contrary to Puertas et al.^28^ a visual screening tool for depression in people living with human immunodeficiency virus (HIV) has demonstrated good psychometric properties in people with high and low levels of education.^29^ However, the tool neglects anxiety symptoms which often coexist with depression. In addition, it focuses on symptoms that often overlap with diabetes. Reddy et al.^27^ argue that symptoms such as low energy complicate the diagnosis of depression in people with diabetes. They further argue that the hospital anxiety and depression scale (HADS) is an appropriate screening tool for depression and anxiety disorders in people with diabetes. However, tools developed in high-income countries written in English for people with higher levels of education might not be appropriate for use in a context such as South Africa, especially in resource-constrained primary health centres.

The identification of patients with mental health problems cannot be successful without culture-fair instruments and instruments free of language bias.

Our study aimed to develop a visual screening tool that can screen both depression and anxiety disorders in people with diabetes and/or hypertension using culture-friendly drawings while avoiding some of the limitations associated with screening tools.

## Methods

### Development of the visual screening tool

**FIGURE 1:**
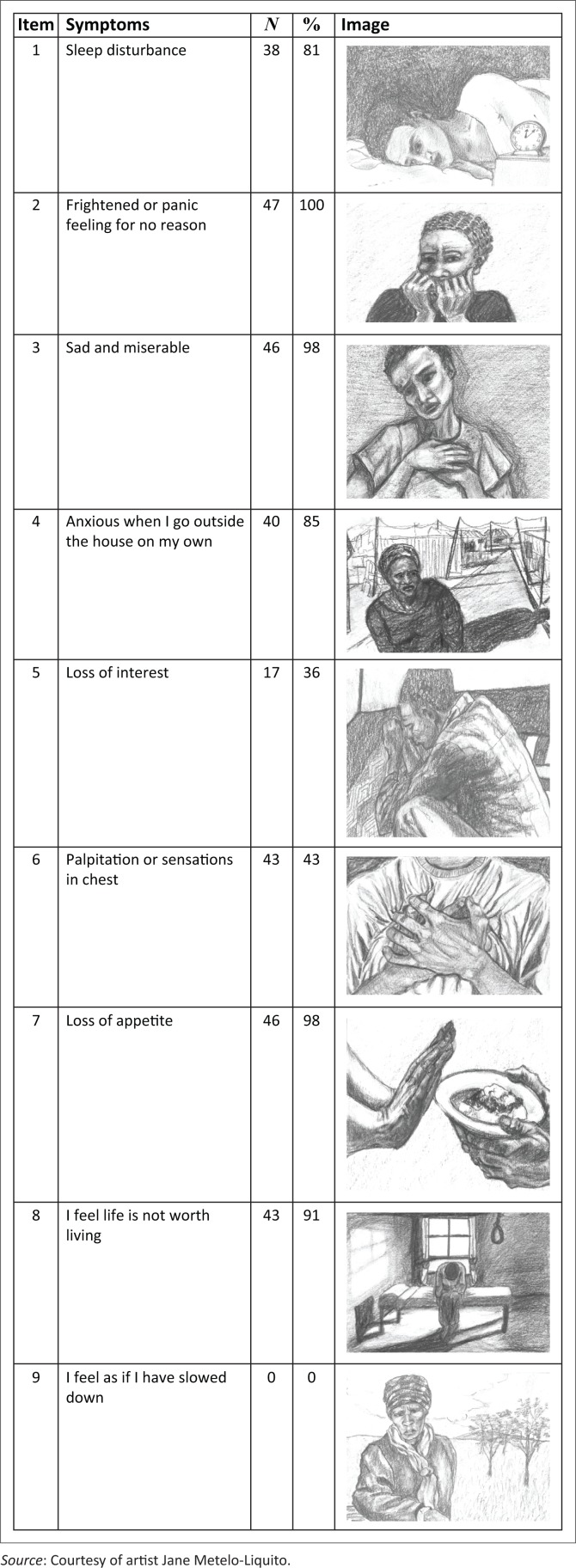

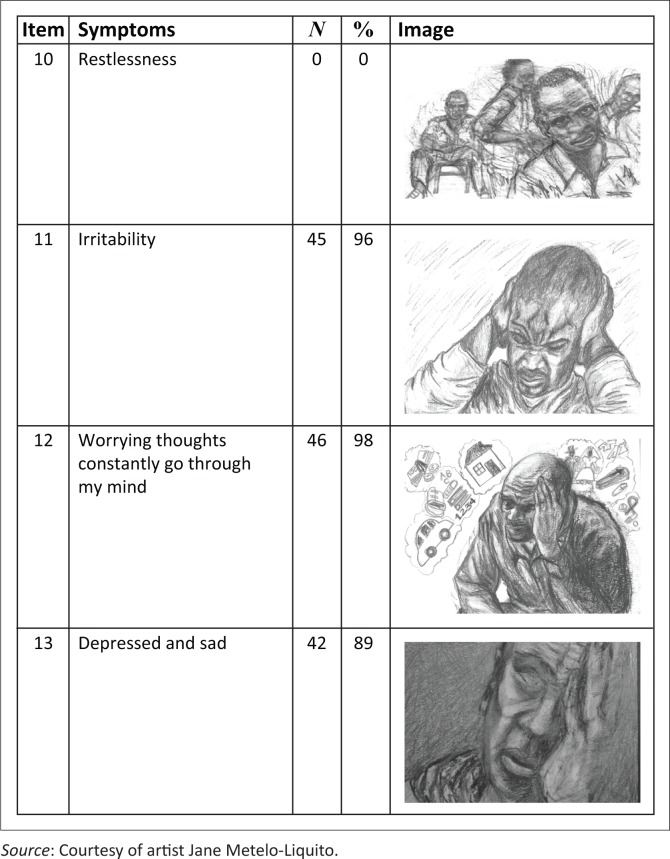
The number of participants describing symptoms associated with depression and anxiety disorders.

As a starting point, an artist, Mrs Jane Metelo-Liquito (Bachelor of Arts Honors, CDAT-I) was asked to make drawings depicting depression and anxiety symptoms before the recruitment for the study began. The drawings were based on the HADS^30^ which has been recommended for screening for depression and anxiety disorders in patients diagnosed with diabetes.^27^ Figure 1 shows the depression and anxiety items included in the development of the visual screening tool. Each item consisted of two drawings: one drawing depicting an abnormal state and the other depicting a normal state. Most of the drawings used in the development of the visual screening tool focused more on the face. The face, according to Betts,^31^ is predominantly associated with the expression of emotions and individual identity.

### Setting

The study was conducted in two not-for-profit organisations in Cape Town and two public health facilities. The not-for-profit organisations included a church in a predominantly white community and an organisation working with business and South African youth in predominantly black and mixed-race communities. The two public health facilities included a maternal mental health clinic unit and a primary health care centre in the Eastern Cape Province of South Africa. The multiple settings included in the study serve multicultural groups from urban and rural communities and this served to minimise bias towards a specific language and/or cultural group. South Africa is a diverse country with the majority of South Africans being black people followed by mixed race people, Indian people, Asian people and white people,^32^ and the most spoken languages are iSizulu, isiXhosa, Afrikaans and English out of the 11 official languages.

### Study design

This was a qualitative cross-sectional study that utilised semi-structured interviews to develop depression and anxiety disorder items for a new visual screening tool.

### Sampling strategy

Purposive sampling was utilised to recruit participants. Individuals were recruited while they were at the settings for a routine scheduled visit. The purpose of the study was explained, and individuals who expressed an interest to take part in the study were seen in a private room. The target study population excluded people with visual and hearing impairment and individuals with intellectual disability based on self-report.

Methodologists such as Morse^33^ recommend 30–60 interviews when conducting semi-structured interviews. This is in agreement with our initial sample size calculation of 60 (at a 95% confidence interval and a precision of 12.75). However, according to Mason,^34^ the most common sample sizes are between 20 and 30 as the necessary information is easily obtained in the interviews and thus fewer participants are needed.^33^ This was also true for our study as themes were already established in a sample size of 47 and recruitment could be terminated after 3 months.

### Data collection

Data were collected over a period of 3 months through semi-structured interviews conducted by the principal researcher who is a clinical psychologist. The structured interviews were conducted in English and Xhosa. English-speaking participants were interviewed in English and Xhosa-speaking participants were interviewed in Xhosa. The demographic questionnaire was administered with all the participants after the completion of written informed consent. This questionnaire was utilised to collect socio-demographic variables such as age, race, sex, marital status, level of education, employment status, family income and medical conditions. Participants were then asked to describe the emotions and thoughts depicted in the 13 drawings, with each interview lasting between 20 and 50 min. The responses provided by the participants were captured on a spreadsheet designed by a statistician.

### Data analysis

The data were analysed through content analysis by coding the obtained data for words relating to symptoms of depression and anxiety disorders. Content analysis begins with predefined categories.^35^ The predefined categories (Figure 1) in this study were based on the HADS. Themes were identified from the descriptions provided by the participants and matched against the HADS items. The number of instances in which participants provided descriptions in response to emotions and thoughts depicted in the drawings was counted.^36^ The meaning of the themes was interpreted, looking for correlation with the symptoms of depression and anxiety disorder as depicted in the drawings.

Trustworthiness of the obtained data was established through respondent validation.^37^ This was done during the interview and at the end of the interview where the principal researcher restated, questioned and summarised the responses given by the participants. This allowed the participants an opportunity to affirm or reject the responses.^38^

Descriptive statistics was used to describe demographic data. Demographic data was summarised as frequencies, percentages, means with standard deviation (SD). STATA version 14 was used for descriptive statistics.

## Ethical considerations

Ethical approval was obtained from the Human Research Ethics Committee of the Faculty of Medicine and Health Sciences at the University of Stellenbosch (Reference number: S14/11/262) and permission was obtained from the Western Cape Department of Health and the Eastern Cape Department of Health to conduct the study. The not-for-profit organisations also granted permission to conduct the study.

## Results

Forty-seven participants participated in the development of the new visual screening tool for anxiety disorders and depression (VISTAD). The mean age of the participants was 34 with SD 12.46 (age range 18–60 years). Thirty-five (74%) of the 47 participants were unemployed and 12 (26%) were employed. The mean income per month was US $487,70 with minimum salary less than US $357,57 and maximum US $4290,84 per month. Table 1 provides a descriptive overview of the demographics of the study participants.

**TABLE 1:** Description of the participants.

Variables	*N*	%
**Gender**		
Female	31	66
Male	16	34
**Race**		
Black people	32	68
Mixed race people	11	23
White people	4	9
**Language**		
isiXhosa	30	64
Afrikaans	14	30
English	1	2
Sotho	1	2
isiZulu	1	2
**Education†**		
No schooling	2	4
Primary schooling (Grade 1 and Grade 6)	14	31
Senior schooling (Grade 7 and Grade 11)	8	18
Matric	13	29
Post-matric qualification	8	18
**Employment**		
Employed	12	26
Unemployed	35	74

*N*, Number of participants; std dev, standard deviation.

† , There were two missing observations on education variable.

Our study determined whether participants identified and described symptoms associated with depression and anxiety disorders correctly. Drawings that depicted symptoms of depression were easily identified and described by the participants. Items 1, 2, 6 and 7 recorded some of the highest frequencies in correct descriptions as symptoms of depression. The number of participants (*N*) who described symptoms of depression and anxiety disorders is presented in Figure 1.

As shown in Figure 1, 38 (81%) participants described item 1 as sleeping disturbance. This description was observed across race, gender and different levels of education. The other nine participants (19%) described item 1 as depicting sadness and depression. Some participants remarked that this is how they were when they were stressed, had too much on their minds and, as a result, struggled to sleep. Item 3 was described as depression, sadness and misery by 46 (98%) participants.

Similar to depression items, anxiety items were described correctly as depicting symptoms associated with anxiety disorders. For example, item 2 ‘frightened or panic feeling for no reason’, was described as anxiety by all the 47 (100%) participants providing accurate descriptions.

The participants’ personal narratives were evident in items 2 and 3. Nine participants (19%) reflected on being diagnosed with HIV and associated anxiety symptoms. The participants responded in the first person when describing the emotions and thoughts depicted in the drawings. Also, it was observed that participants became emotional and tearful during the interviews. There were 4 participants who became tearful during the interviews. In responding to item 3, sad and miserable, participants related narrative life stories which were characterised by sadness. Item 4, ‘anxious when outside on my own’, included ‘scared, hyperalert, fear of being attacked, fearful, cannot defend herself, shadow implies fear, abused, unsafe’. Participants with varying levels of education, different races and languages were able to understand the drawings and provided descriptions of symptoms associated with anxiety and depression.

Thirty participants (64%) described item 9 as portraying sadness and loneliness in a troubled person, and descriptions such as the person was praying were also mentioned. Some of the participants described the feeling of slowing down as a story of a depressed family member (item 9). Furthermore, participants empathised with the woman on the drawing reporting she was confused, on her own and lived below the poverty line (item 9). The feeling of slowing down (item 9) and restlessness (item 10) were not identified correctly by all the participants. Seven participants (15%) narrated their life stories in relation to the feeling of slowing down (item 9) and feeling restlessness (item 10). These stories were characterised by depression and anxiety. The picture depicting restlessness (item 10) was found to be the most unclear item. This was described as a group of angry people, poverty-stricken individuals, prisoners and sad individuals.

Participants expressed sadness at the person depicted in item 13, stating that the person was stressed and had many life problems. Some of the participants personally identified with the emotional state depicted in item 13. These participants began to describe issues that concerned them in life. The descriptions provided by the participants with varying levels of education and different races and languages were associated with symptoms of depression and anxiety as predefined in the HADS, and this showed that the items could be considered for inclusion in the visual screening tool.

The items that were frequently described correctly included depression items: sleep disturbance, feeling miserable and sad, appetite, feeling life is not worth living, and anxiety items; feeling frightened or having panic feelings for no reason, feeling frightened when going out of the house on my own, getting palpitations or sensations ‘butterflies’ in stomach or chest, irritable than usual and worrying thoughts. Items that were least understood by the participants and did not match the predefined categories were removed, both the normal and abnormal state drawings.

## Discussion

Forty-seven participants participated in the development of the VISTAD. The study confirmed that cultural and educational background had no bearing on the ability of participants to identify and describe symptoms associated with depression and anxiety disorders. Participants with low levels of education performed similarly to those with higher levels of education, and this was observed across cultures. Previous studies found visual screening tools to be valid for use as screening tools in people with low and high levels of education.^29^ The participants identified symptoms associated with depression and anxiety disorders accurately. These findings are consistent with observations made by Vick and Strauss.^39^ Also, when providing descriptions, participants gave reasons why people felt and thought the way did. For example, when the sleep disturbance item was identified, participants described reasons why the person on the drawing was not sleeping. Furthermore, descriptions of the items were connected to personal narratives of the participants. Drawings, according to Oster and Crone,^40^ are often viewed as less threatening than direct verbal interaction.

The participants used their life stories in describing emotions and thoughts depicted in the drawings. The use of drawings potentially offers invaluable opportunities to explore emotional status across cultural boundaries. Similar to the Rorschach technique, individuals have tremendous freedom of response.^41^ Individuals can report on what they are seeing instead of offering an either true or false response to a written statement.^41^ The use of drawings depicting emotions can provide access to the emotions of patients suffering from chronic medical conditions. This study lays the groundwork for studying the ability of the drawings as a projective tool. The assumption of projective instruments is that the respondent will project onto an image, thus expressing unconscious needs that he or she is ordinarily unable or unwilling to report.^42^

The participants often responded in the first person and added statements such as ‘that was me’ when they identified with a specific drawing, such as sleep disturbance item. Projective drawings provide information that can enrich an individual’s description of his or her own experience.^43^ A projective test involves, amongst other stimuli, a drawing chosen because it will elicit hidden meanings and not because the tester has preconceived ideas of what it should mean.^43^ In our study we held preconceived ideas about what the drawings mean as they were based on the HADS. However, it was not expected that participants would give personal stories in their responses to the drawings. The descriptions of the emotions and thoughts were accompanied by narratives offering unique material for understanding the participants’ life stories.

The new screening tool was named the VISTAD. Of the 13 drawings depicting symptoms associated with depression and anxiety disorders, we selected 10 to be used in the validation study of the VISTAD. These included the normal state drawings. Items: ‘feeling as if one’s mind has slowed down’, ‘restlessness’ and ‘loss of interest’ were excluded from the validation study. This included both the drawings portraying a normal and an abnormal state. The VISTAD will be validated against the mini international neuropsychiatric interview (MINI) for accuracy as a screening tool in a primary health care population diagnosed with hypertension or diabetes.

### Limitations

The research participants were predominantly of a low socio-economic status. However, the VISTAD is designed for primary health care, and primary health care predominantly services people of low socio-economic status. Furthermore, the study did not include all racial groups. There were no Indian participants or Asian participants. The answers provided by the participants were captured on a spreadsheet during the interview and were not recorded as the participants described emotions and thoughts.

## Conclusions

The findings of this study demonstrate that the drawings are appropriate for use with people from different cultural backgrounds and varying educational levels to describe the same feelings and thoughts. As a next step, in order to establish the psychometric properties of the VISTAD, it should be validated against the MINI in a primary health care population with hypertension or diabetes.
